# Development of a High-Titer Culture Medium for the Production of Cholesterol by Engineered *Saccharomyces cerevisiae* and Its Fed-Batch Cultivation Strategy

**DOI:** 10.4014/jmb.2106.06026

**Published:** 2021-12-01

**Authors:** Ling-Xu Wang, Gao-Fan Zheng, Xiu-Juan Xin, Fa-Liang An

**Affiliations:** State Key Laboratory of Bioreactor Engineering, East China University of Science and Technology, Shanghai 200237, P. R. China

**Keywords:** Cholesterol, *S. cerevisiae*, fermentation optimization, fed-batch culture, citric acid

## Abstract

Steroids are a class of compounds with cyclopentane polyhydrophenanthrene as the parent nucleus, and they usually have unique biological and pharmacological activities. Most of the biosynthesis of steroids is completed by a series of enzymatic reactions starting from cholesterol. Synthetic biology can be used to synthesize cholesterol in engineered microorganisms, but the production of cholesterol is too low to further produce other high-value steroids from cholesterol as the raw material and precursor. In this work, combinational strategies were established to increase the production of cholesterol in engineered *Saccharomyces cerevisiae* RH6829. The basic medium for high cholesterol production was selected by screening 8 kinds of culture media. Single-factor optimization of the carbon and nitrogen sources of the culture medium, and the addition of calcium ions, zinc ions and citric acid, further increased the cholesterol production to 192.53 mg/l. In the 5-L bioreactor, through the establishment of strategies for glucose and citric acid feeding and dissolved oxygen regulation, the cholesterol production was further increased to 339.87 mg/l, which was 734% higher than that in the original medium. This is the highest titer of cholesterol produced by microorganisms currently reported. The fermentation program has also been conducted in a 50-L bioreactor to prove its stability and feasibility.

## Instruction

Steroids are compounds with cyclopentane phenanthrene as the parent nucleus. Their unique biological structure usually brings rich biological and pharmacological activities, especially of the antibacterial and antiviral type [1], [2]. Steroids are widely used in making immunity inhibitors, hormone drugs, diuretics, cardio tonics, etc. Hydrocortisone, progesterone, diosgenin and dexamethasone are all commonly used clinically steroid drugs [3]. Cholesterol was the first type of steroid discovered. As an essential element of the cell membrane, cholesterol plays an important role in maintaining membrane fluidity, cell growth and proliferation [4]. At the same time, cholesterol is also an important raw material and intermediate for the synthesis of most steroids like bile acids, vitamin D, and some steroid hormones [5]. Biotransformation is more advantageous to the synthesis of some complex or structure-specific compounds, and compared with the traditional preparation methods, the conditions of biotransformation are milder and more environmentally friendly, and most organisms are able to carry out the transformation of steroids [6]. Therefore, in recent years, cholesterol studies looking to obtain active steroids through microbial transformation have been extensive, especially in the side chain cleavage, hydroxylation, dehydrogenation, reduction, isomerization, and esterification reactions of cholesterol [5, 7, 8]. Studies have shown that cytochrome P450 enzymes play a key role in cholesterol synthesis and metabolism [9]. These studies also demonstrated the feasibility of using industrial microorganisms to synthesize valuable steroids. Due to the difficulty of screening strains and enzymes, researchers tend to construct industrial microorganisms to biosynthesize steroids from basic nutrient materials. Therefore, it is necessary to construct an engineered strain to produce cholesterol first.

**Fig. 1:**
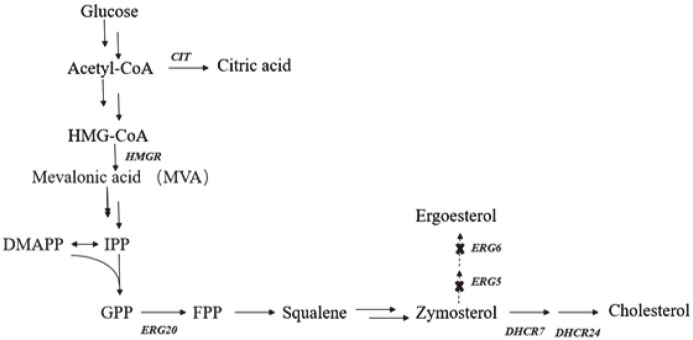
Biosynthesis of cholesterol from glucose in *S. cerevisiae* RH6829.

*S. cerevisiae* is one of the most widely used engineering microorganisms. Compared with prokaryotes, it has a complex downstream structural modification system. Moreover, *S. cerevisiae* itself has a sterol metabolism pathway, and the end product of metabolism is ergosterol [10], which is the fundamental reason why *S. cerevisiae* is employed as a steroid-producing strain for research [11]. Szczebara *et al*. introduced 13 genes related to the expression of 8 mammalian proteins into *S. cerevisiae*, optimized two mitochondrial systems and removed harmful side reactions, and successfully achieved the total synthesis of hydrocortisone from glucose in recombinant *S. cerevisiae* [12]. Xia *et al*. achieved the biosynthesis of lycopene in *S. cerevisiae*, and through citric acid fed-batch fermentation, the titer of lycopene was increased to 115.64 mg/l [13]. Through inactivating the C-22 sterol dehydrogenase (ERG5) and C24-methyltransferase genes (ERG6) in *S. cerevisiae*, and introducing C-8 sterol isomerase (ERG2), C-5 sterol desaturase (ERG3) and C24-reductase gene (DHCR24) from mice and vertebrates, they successfully synthesized 7-dehydrocholesterol in *S. cerevisiae* [14, 15]. Subsequently, Guo Xiaojing *et al*. further increased the production of 7-dehydrocholesterol to 1.07 g/l in the 5-L bioreactor by overexpressing a series of genes in the mevalonate pathway [16]. By introducing sterol C-7 reductase and sterol C-24 reductase from different sources, Cleiton M *et al*. constructed a *S. cerevisiae* strain RH6829 that stably produces cholesterol instead of ergosterol, and obtained a cholesterol titer of 1 mg/g·FW [17]. The biosynthetic route of cholesterol is shown in Fig. 1.

Although it is now feasible to use engineered microorganisms to synthesize cholesterol and other steroids converted from it, the production of cholesterol is too low to further produce other high-value steroids from cholesterol as the raw material and precursor. Moreover, there are currently very few reports on the improvement of cholesterol production in engineered microorganisms. Therefore, this study proposed a combinational fermentation strategy of *S. cerevisiae* RH6829 to increase the production of cholesterol as an intermediate in the synthesis of steroids.

## Materials and Methods

### Strain and Media

The strain used in this work is *S. cerevisiae* RH6829, provided by Zhang Yan-Sheng's team from Shanghai University, and stored in 40% (*v/v*) glycerol at -80°C. The components of each medium used in this work are shown in Table 1.

### Culture Condition

Fifty microliters of the preserved *S. cerevisiae* RH6829 was added to a serum bottle containing 5 ml of seed culture medium to prepare the inoculum. The first-stage seed was cultured at 30°C and 175 rpm for 48 h on a shaker. To obtain the second-stage seed, 2 ml of the first-stage seed was added into 50 ml of seed culture medium in a 250-ml shake flask and cultured for 24 h under the same condition. Finally, 5 ml of the second-stage seed was added into 50 ml of various media in 250-ml shake flasks and then fermentation was conducted at 30°C and 175 rpm for 120 h on a shaker. Although the cell growth rate differs in various media, considering the time cost, the cholesterol concentration at 120 h of fermentation was uniformly selected as the reference index. Three biological replicates were conducted for each batch of fermentation.

### Calcium Ion and Zinc Ion Feeding

The concentration of calcium ions in cells will affect the growth of bacteria and various metabolic activities, and zinc ions can effectively improve the ethanol tolerance of *S. cerevisiae* to reduce the toxic effects of ethanol produced during the fermentation process [18]. Therefore, different final concentrations of CaCl_2_ and ZnSO_4_ were added at different times in medium M9 during the shaking flask fermentation process to determine the optimal concentration and addition time of calcium ion and zinc ion.

### Citric Acid Feeding and Measurement of Gene Transcriptions by Real-Time Quantitative PCR (qRT-PCR)

Studies have shown that the addition of citric acid can promote the production of lycopene in engineered *S. cerevisiae* [13], and there are many similar pathways in the synthesis of lycopene and cholesterol, such as the mevalonate pathway. Therefore, different final concentrations of citric acid were added at 0 h and 12 h of fermentation in medium M10 to investigate its effect on cholesterol synthesis.

HMG1, ERG20, DHCR7, DHCR24 were the key enzyme genes in the cholesterol synthesis pathway; CIT1 was the citrate synthase gene, and ACT1 is the gene encoding the actin of *S. cerevisiae*, and is also a commonly used reference gene in PCR experiments to quantitatively characterize the transcription level of other genes. These genes and their primer information are shown in Table 2. The total RNA of *S. cerevisiae* RH6829 was extracted using TriZol solution treated as an extracting solution. RNA concentration was determined using a spectrophotometer at A_260/280_. Reverse transcription was achieved using total RNA as the starting material and the Hifair III 1st Strand cDNA Synthesis SuperMix (Yeasen Biotechnology; Shanghai, China). The method of real-time quantitative PCR was the modified method of Liu *et al*. [19]. *S. cerevisiae* RH6829 with fermentation for 12 h (exponential phase) and 36 h (stationary phase) were selected to investigate the transcription levels of these genes.

### Batch and Fed-Batch Fermentation in 5-L Bioreactor

The optimized strategy of fed-batch fermentation to increase cholesterol production by feeding additional glucose and citric acid was conducted in a 5-L reactor from medium M11, with an inoculum volume of 10%, aeration rate of 3 l/min, and agitation speed of 300 rpm. During the fermentation process, 100 ml of 500 g/l glucose and 8 g/l citric acid were added every 12 h. At the same time, using the reactor's stirring speed-dissolved oxygen joint control function, the following dissolved oxygen control strategy was established: when the dissolved oxygen in the fermentation broth was less than 30%, the speed was gradually increased at a step value of 20 rpm to increase the dissolved oxygen level; when the dissolved oxygen reached 80%, the speed was gradually reduced at the same step value. The lower and upper limits of the speed were set to 200 and 500 ×*g*. Through the joint control of speed-dissolved oxygen, the bacteria were always in a stable environment with sufficient dissolved oxygen. During the first 24 h of the fermentation process, samples were taken every 6 h to detect cholesterol production and biomass, and then every 12 h thereafter. In addition, when citric acid was added, to avoid the influence of pH reduction of the medium caused by its acidity, 6 g/l of CaCO_3_ was added to maintain a stable pH.

### Fed-Batch Fermentation in 50-L Bioreactor

Scale-up fermentation with medium M11 in a 50-L reactor was conducted to verify the stability and feasibility of the fermentation scheme. The inoculation amount was 10%, the aeration rate was 30 l/min, and the agitation speed was 150 rpm. During the fermentation process, 1 L of 500 g/l glucose was added every 12 h. Samples were taken every 12 h to determine the cholesterol production.

## Analytical Methods

### Measurement of Biomass and Residual Glucose

The biomass of strains was characterized by the fresh weight (FW). First, 25 ml of fermentation broth was collected in a centrifuge tube and centrifuged at 3000 ×*g* for 10 min to obtain the cells. The collected cells were then washed with distilled water and centrifuged three times. The biomass was calculated by the weight difference between the centrifuge tube with cells and centrifuge tube. The residual glucose in the broth was measured with an SBA-40E glucose bioanalyzer (Institute of Biology, Shandong Academy of Sciences).

### Cholesterol Extraction and Qualitative Analysis

The cells were resuspended in a mixture of 30 ml ethanol and 10 ml 60% (w/w) KOH, heated at 85°C for 2 h, cooled and extracted twice with 20 ml petroleum ether. The petroleum ether phase was pooled and evaporated to dryness in a rotary evaporator. The product was dissolved and collected in 10 ml methanol.

The cholesterol concentration was determined by HPLC (Agilent Technologies 1260 Infinity, USA) on an ODS column though external standard method (Eclipse Plus C18, 4.6 mm × 250 mm, 5 μm, 1 ml/min methanol as mobile phase) and the ultraviolet detection was at 205 nm. The standard curve : Y = 9.3689X + 58.362 (*R*^2^ = 0.9994, linearity range : 31.25-1,000 mg/l. The formulation of the standard curve was based on the corresponding relationship between the peak area of the cholesterol standard and its concentration. The cholesterol concentration of the sample was calculated according to the standard curve based on its peak area.

## Results and Discussion

The media (M1-M8) used in this study are commonly used in yeast culture and production of secondary metabolites in our laboratory. The single-factor investigation of the added concentration and time of the nutrient elements in the selected basal medium was done to obtain the optimal conditions.

### Medium Optimization for High Production of Cholesterol

**Fig. 2:**
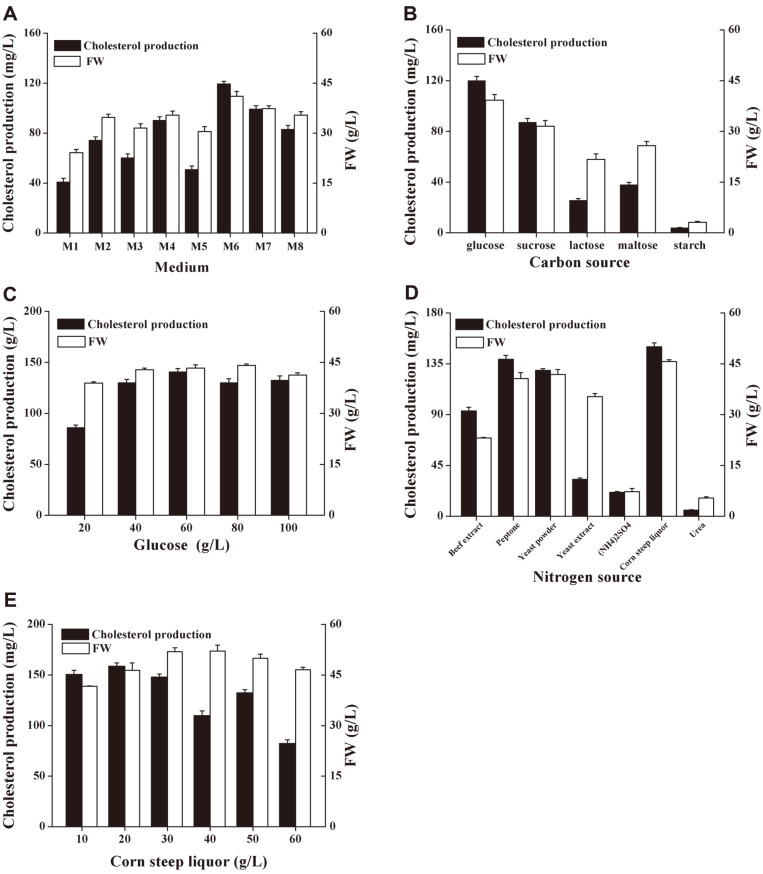
Screening of basic medium for cholesterol production and optimization of carbon and nitrogen sources in medium M6. (**A**): Cholesterol production and yeast biomass in medium M1-M8; (**B**): Cholesterol production and biomass in medium M6 under different carbon sources, and the concentration of each carbon source was 80 g/l; (**C**): Cholesterol production and biomass in medium M6 under different glucose concentrations; (**D**): Cholesterol production and biomass in medium M6 under different nitrogen sources, and the concentration of each nitrogen source was 20 g/l; (**E**): Cholesterol production and biomass in medium M6 under different corn steep liquor concentrations. All experiments were repeated three times independently.

Eight different media were selected to screen out the basic media that can provide high production of cholesterol. As shown in the Fig. 2A, medium M6 has the highest cholesterol titer, so it was selected for subsequent single-factor optimization of carbon and nitrogen sources and further optimization. Figs. 2B and 2C showed that the most suitable carbon source for cholesterol production in the culture medium is 60 g/l glucose, while Figs. 2D and 2E show that the most suitable nitrogen source is 20 g/l corn steep liquor. Through this step, we obtained the new medium M9 (glucose 60 g/l, corn steep liquor 20 g/l, K_2_HPO_4_ 3 g/l, MgSO_4_ 1.5 g/l) for subsequent research.

**Fig. 3:**
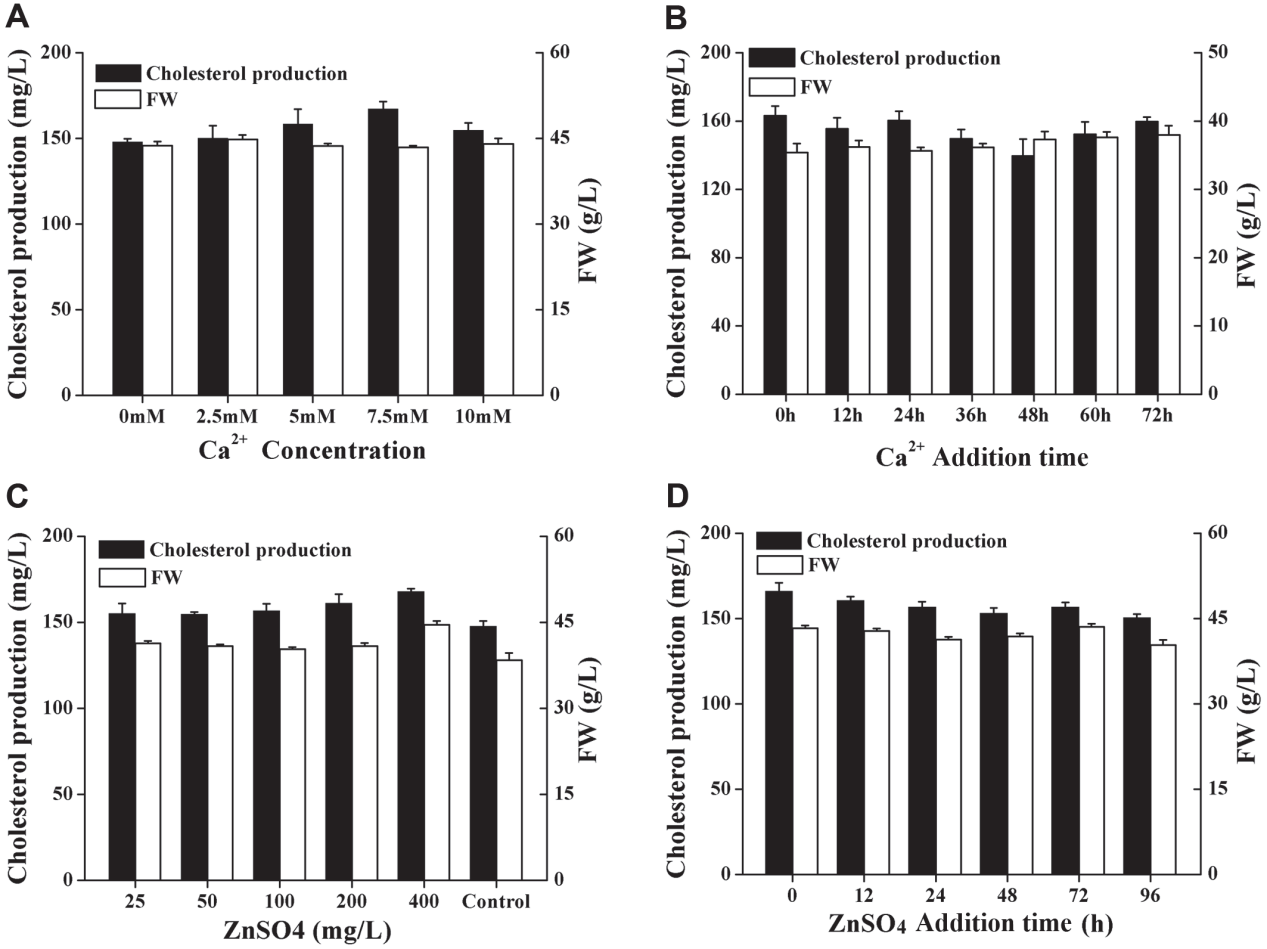
Fermentation under the addition of calcium ions and zinc ions. (**A**): Cholesterol production and biomass in medium M9 with different concentrations of CaCl_2_ added at the beginning of fermentation; (**B**): Cholesterol production and biomass in medium M9 with the addition of 5 mM CaCl_2_ at different times; (**C**): Cholesterol production and biomass in medium M9 with different concentrations of ZnSO_4_ added at the beginning of fermentation; (**D**): Cholesterol production and biomass in medium M9 with the addition of 400 mg/l ZnSO_4_ at different times. All experiments were repeated three times independently.

Different concentrations of CaCl_2_ from 0 to 10 mM and different concentrations of ZnSO_4_ from 25 to 400 mg/l were added to the medium M9 to investigate their optimal concentration and time. Figs. 3A and 3B showed that the most suitable calcium ions addition strategy for cholesterol production is adding 7.5 mM CaCl_2_ at the beginning of fermentation. Figs. 3C and 3D showed that the most suitable zinc ion addition strategy for cholesterol production is adding 0.4 g/l ZnSO_4_ at the beginning of fermentation. Through this step, we obtained the new medium M10 (glucose 60 g/l, corn steep liquor 20 g/l, K_2_HPO_4_ 3 g/l, MgSO_4_ 1.5 g/l, CaCl_2_ 0.83 g/l, ZnSO_4_ 0.4 g/l) for subsequent research.

### Effect of Adding Citric Acid on Cholesterol Production

**Fig. 4:**
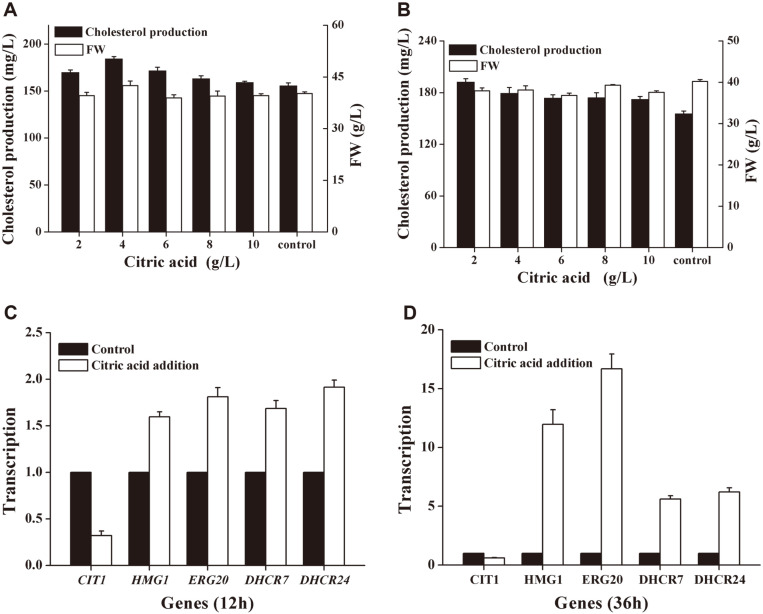
Effects of citric acid addition on cholesterol production and the transcription level of related genes. (**A**): Cholesterol production and biomass in medium M10 with different concentrations of citric acid added at the beginning of fermentation; (**B**): Cholesterol production and biomass in medium M10 with different concentrations of citric acid added at 12 h after fermentation; (**C**): The transcription level of each gene at 12 h after fermentation with the addition of citric acid, the y-axis label "Transcription" indicates the relative fold change of the expression of each gene between the experimental group and the control group; (**D**): The transcription level of each gene at 36 h after fermentation with the addition of citric acid, the y-axis label "Transcription" indicates the relative fold change of the expression of each gene between the experimental group and the control group. All experiments were repeated three times independently.

Different concentrations of citric acid were added at the beginning and at 12 h of fermentation in medium M10. Figs. 4A and 4B showed that adding a certain concentration of citric acid at the beginning of fermentation or at 12 h of fermentation promote the synthesis of cholesterol. Adding 4 g/l of citric acid at the beginning of fermentation has the most obvious promotion effect on cholesterol synthesis, and the cholesterol production was 192.53 mg/l, which was 21.23% higher than that without adding citric acid. Through this step, we obtained the new medium M11 (glucose 60 g/l, corn steep liquor 20 g/l, K_2_HPO_4_ 3 g/l, MgSO_4_ 1.5 g/l, CaCl_2_ 0.83 g/l, ZnSO_4_ 0.4 g/l, citric acid 4 g /l) for subsequent research.

In Figs. 4C and 4D, whether in the exponential phase or the stationary phase, the addition of citric acid reduces the transcription level of the citrate synthase gene (CIT1) of the bacteria itself. On the contrary, the transcription level of these genes (HMG1, ERG20, DHCR7, DHCR24) in the cholesterol synthesis pathway has increased, especially in the stationary phase, so this effect is more significant. Among them, the expression of ERG20 increased by 16.69 times after 36 h of fermentation.

As shown in Fig. 1, citric acid, one of the components of the tricarboxylic acid cycle, is synthesized from acetyl-CoA and oxaloacetate. Based on the results of the gene transcription level determination experiment in Fig. 4, the principle of the increase in cholesterol production caused by the addition of citric acid can be preliminarily analyzed: the addition of citric acid reduces the gene transcription level of its own citrate synthase and hindered the synthesis of citric acid, therefore, the raw material acetyl-CoA can accumulate and enter into the mevalonate pathway to synthesize more cholesterol.

### Batch and Fed-Batch Fermentation in 5-L & 50-L Bioreactors

**Fig. 5:**
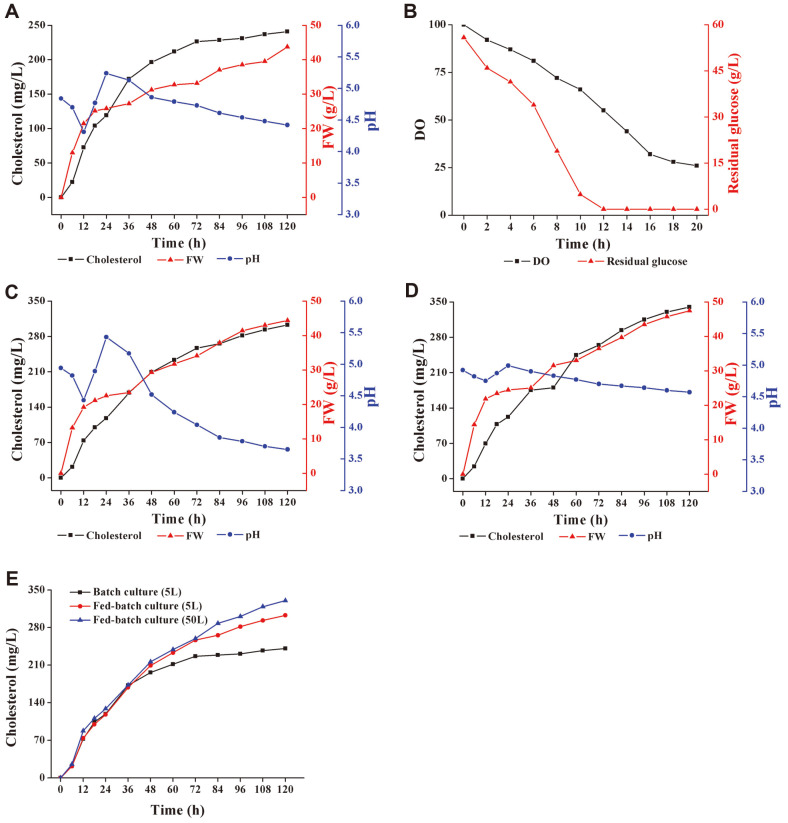
Batch fermentation and fed-batch fermentation in 5-L & 50-L bioreactor. (**A**): Batch fermentation of medium M10 in a 5-L bioreactor; (**B**): Dissolved oxygen and glucose consumption in the first 20 h during batch fermentation; (**C**): Fed-batch fermentation of medium M10 in a 5-L bioreactor, 100 ml of 500 g/l glucose was added every 12 h; (**D**): Fed-batch fermentation of medium M10 in a 5-L bioreactor, 100 ml of 500 g/l glucose and 8 g/l citric acid were added every 12 h, as well as the stirring speed-dissolved oxygen joint control was conducted; (**E**): Fed-batch fermentation of medium M10 in a 50-L bioreactor, 1 L of 500 g/l glucose was added every 12 h.

Figs. 5A and 5B showed that in batch fermentation, along with the rapid consumption of dissolved oxygen, the residual glucose in the broth decreased to 0 after 12 h of fermentation. Therefore, the follow-up feeding experiment was started after 12 h of fermentation. At the end of batch fermentation, the cholesterol titer obtained was 240.87 mg/l. Fig. 5C showed that after adding glucose every 12 h, the cholesterol production and biomass during the fermentation process were significantly increased. Meanwhile, glucose metabolism brought greater pH changes in the broth. By the end of fed-batch fermentation, the cholesterol titer obtained was 302.72 mg/l, which was 25.68% higher than that of batch fermentation. Fig. 5D showed that when both glucose and citric acid were added every 12 h, the cholesterol production in the fermentation process is further improved. The presence of CaCO_3_ made the pH of the broth more stable. At the end of fed-batch fermentation, the cholesterol titer obtained was 339.87 mg/l, which was 41.10% higher than that of batch fermentation. Fig. 5E showed that in the glucose fed-batch fermentation in the 50-L bioreactor, the cholesterol production reached 330.11 mg/l, which was 9.05%higher than that in the 5-L reactor under the same conditions. The successful scale-up of the fermentation proved that the fermentation scheme is stable and reliable.

In this paper, we developed a high-titer culture medium for the production of cholesterol by *S. cerevisiae* RH6829 through basic medium selection, optimization of carbon and nitrogen sources, and investigation of additives. The fed-batch of glucose and citric acid and the regulation of dissolved oxygen were established on a 5-L reactor, which further increased the maximum cholesterol production to 339.87 mg/l. Moreover, the scale-up of glucose fed-batch fermentation was successfully completed in a 50-L bioreactor. This work provides theoretical support for the production of more high-value steroids using cholesterol as a raw material and precursor.

## Figures and Tables

**Table 1:** Composition of each medium used in this study.

Medium	Composition (g/l)
Seed medium	Glucose 20, Yeast Extract 10, Peptone 20
M1 (Original medium)	Same as the seed medium
M2	Glucose 50, Yeast Powder 10, (NH_4_)_2_SO_4_ 15, K_2_HPO_4_ 2
M3	Sucrose 91.8, (NH_4_)_2_SO_4_ 10.3, KH_2_PO_4_ 8.7, MgSO_4_ 1.5
M4	Glucose 40, Sucrose 20, Peptone 10, Yeast Extract 10, (NH_4_)_2_SO_4_ 5, KH_2_PO_4_ 1, NaCl 1, MgSO_4_ 5, CaCl_2_ 0.1, NaNO_3_ 2
M5	Glucose 20, (NH_4_)_2_SO_4_ 5, Peptone 12,5, KH_2_PO_4_ 9, MgSO_4_ 1, NaCl 0.2, ZnSO_4_ 0.01
M6	Glucose 80, Beef Extract 20, K_2_HPO_4_ 3, MgSO_4_ 1.5
M7	Glucose 53.4, Peptone 21.5, Yeast Extract 10, K_2_HPO_4_ 2.98
M8	Sucrose 40, Peptone 30, KH_2_PO_4_ 1, NaCl 1
M9	Glucose 60, Corn steep liquor 20, K_2_HPO_4_ 3, MgSO_4_ 1.
M10	Glucose 60, Corn steep liquor 20, K_2_HPO_4_ 3, MgSO_4_ 1.5, CaCl_2_ 0.83, ZnSO_4_ 0.4
M11	Glucose 60, Corn steep liquor 20, K_2_HPO_4_ 3, MgSO_4_ 1.5, CaCl_2_ 0.83, ZnSO_4_ 0.4, Citric acid 4

**Table 2:** Genes and primer sequences for qRT-PCR.

Target gene	Primer name	Primer sequence (5′-3′)
CIT1	CIT1 - forward CIT1 - reverse	TGGCCCATTACATGGTCGTG AACAACTCTCCCTGCGTTCA
HMG1	HMG1 - forward HMG1 - reverse	ATACTACGAGAGCGGTTGCG ACAACAAGCGCCAAATACGC
ERG20	ERG20 - forward ERG20 - reverse	AAGGACTCAGTCGCAGAAGC CCACGAGACTCATCGACCTG
DHCR7	DHCR7 - forward DHCR7 - reverse	TCATAACCCGCGTCATAGCC GCGGGGAAACCTTCACAAAC
DHCR24	DHCR24 - forward DHCR24 - reverse	CCAACAAGGCAGAGTGTTGC ACTGGTGTGCTCTTCTTCCG
ACT1	ACT1 - forward ACT1 - reverse	GAAATGCAAACCGCTGCTCA TACCGGCAGATTCCAAACCC

## References

[1] Fernandes P, Cruz A, Angelova B, Pinheiro HM, Cabral JMS (2003). Microbial conversion of steroid compounds: recent developments. Enzyme Microb. Technol..

[2] Sultana N (2018). Microbial biotransformation of bioactive and clinically useful steroids and some salient features of steroids and biotransformation. Steroids.

[3] Chen Y, Tang Y-M, Yu S-L, Han Y-W, Kou J-P, Liu B-L (2015). Advances in the pharmacological activities and mechanisms of diosgenin. Chinese J. Nat. Med..

[4] Bhatti HN, Khera RA (2012). Biological transformations of steroidal compounds: a review. Steroids.

[5] Giorgi V, Menendez P, Garcia-Carnelli C (2019). Microbial transformation of cholesterol: reactions and practical aspects-an update. World J. Microbiol. Biotechnol..

[6] Parshikov IA, Sutherland JB (2015). Biotransformation of steroids and flavonoids by cultures of *Aspergillus niger*. Appl. Biochem. Biotechnol..

[7] Thomas ST, VanderVen BC, Sherman DR, Russell DG, Sampson NS (2011). Pathway profiling in *Mycobacterium tuberculosis*: elucidation of cholesterol-derived catabolite and enzymes that catalyze its metabolism. J. Biol. Chem..

[8] Wang ZF, Huang YL, Rathman JF, Yang S-T (2002). Lecithin-enhanced biotransformation of cholesterol to androsta-1,4-diene-3,17-dione and androsta-4-ene-3,17-dione. J. Chem. Technol. Biotechnol..

[9] McLean KJ, Hans M, Munro AW (2012). Cholesterol, an essential molecule: diverse roles involving cytochrome P450 enzymes. Biochem. Soc. Trans..

[10] Hu Z, He B, Ma L, Sun Y, Niu Y, Zeng B (2017). Recent advances in ergosterol biosynthesis and regulation mechanisms in *S. cerevisiae*. Indian J. Microbiol..

[11] Nagegowda DA, Gupta P (2020). Advances in biosynthesis, regulation, and metabolic engineering of plant specialized terpenoids. Plant Sci..

[12] Szczebara FM, Chandelier C, Villeret C, Masurel A, Bourot S, Duport C (2003). Total biosynthesis of hydrocortisone from a simple carbon source in yeast. Nat. Biotechnol..

[13] Li X, Wang Z, Zhang G, Yi L (2019). Improving lycopene production in *S. cerevisiae* through optimizing pathway and chassis metabolism. Chem. Eng. Sci..

[14] Hohmann HP, Lehmann M (2012). Production of non-yeast sterols by yeast.

[15] Lang C, Markus V (2011). Preparation of 7-dehydrocholesterol and/or the biosynthetic intermediates and/or secondary products thereof in transgenic organisms.

[16] Guo XJ, Xiao WH, Wang Y, Yao MD, Zeng BX, Liu H (2018). Metabolic engineering of *S. cerevisiae* for 7-dehydrocholesterol overproduction. Biotechnol. Biofuels.

[17] Souza CM, Schwabe TM, Pichler H, Ploier B, Leitner E, Guan XL (2011). A stable yeast strain efficiently producing cholesterol instead of ergosterol is functional for tryptophan uptake, but not weak organic acid resistance. Metab. Eng..

[18] Cheng C, Zhang M, Xue C, Bai F, Zhao X (2017). Development of stress tolerant *S. cerevisiae* strains by metabolic engineering: New aspects from cell flocculation and zinc supplementation. J. Biosci. Bioeng..

[19] Li S, Liu L, Chen J (2015). Mitochondrial fusion and fission are involved in stress tolerance of *Candida glabrata*. Bioresour. Bioprocess..

